# USE OF DECELLULARIZED HUMAN AMNIOTIC MEMBRANE IN INTESTINAL ANASTOMOSES: A STUDY IN RATS TREATED WITH 5-FLUOROURACIL

**DOI:** 10.1590/0102-6720202400049e1843

**Published:** 2024-12-16

**Authors:** Daniel Dantas FERRARIN, Osvaldo MALAFAIA, Nicolau Gregori CZECZKO, Luiz Fernando KUBRUSLY, Marcos Fabiano SIGWALT, Eros Luiz de SOUSA, João Carlos Domingues REPKA, Pedro Henrique Lambach CARON

**Affiliations:** 1 Faculdade Evangélica Mackenzie – Curitiba (PR), Brazil;; 2 Hospital e Maternidade Angelica Caron – Campina Grande do Sul (PR), Brazil.

**Keywords:** Amnion, Surgical Anastomosis, Drug Therapy, Combination, Membranes, Âmnio, Anastomose Cirúrgica, Quimioterapia Combinada, Membranas

## Abstract

**BACKGROUND:**

Nowdays, more relevant applications of perinatal derivatives, such as amniotic membrane (AM), are emerging in our environment as a source of biomaterials for use in different healing processes. The study of anastomosis healing associated with antimetabolic drugs such as 5-fluorouracil (5-FU) is a potential target of AM.

**AIMS:**

To evaluate the healing effects of AM in rats treated with 5-FU at a dose of 20 mg/kg on the seventh day of postoperative evolution, regarding the parameters percentage of type I collagen (mature), cell viability, microvascular density and formation of granulation tissue.

**METHODS:**

Thirty-two Wistar rats were used, submitted to colotomy and colorraphy, separated into four groups of eight, which received different treatments daily, intraperitoneally, until the day of sacrifice: saline solution (C), 20 mg/kg 5-FU, 20 mg/kg 5-FU and AM.

**RESULTS:**

Treatment with 20 mg/kg of 5-FU, on the seventh postoperative day, induced adverse effects on the anastomotic healing process, evidenced by a decrease in the percentage of type I (mature) collagen, cell viability, microvascular density, fibrin-leukocyte scab formation and angiofibroblast proliferation; the use of AM under these conditions induced an improvement in the percentage of type I (mature) collagen.

**CONCLUSIONS:**

Treatment with 20 mg/kg of 5-FU on the seventh postoperative day induced adverse effects on the anastomotic healing process, and the use of AM under these conditions induced an improvement in the percentage of type I (mature) collagen.

## RESUMO

**RACIONAL:**

Atualmente emergem em nosso meio aplicações mais relevantes dos derivados perinatais, por exemplo a membrana amniótica (MA), como fonte de biomateriais para emprego em diferentes processos cicatriciais. O estudo de cicatrização de anastomoses associadas a drogas antimetabólicas como o 5-fluorouracil (5- FU) é um potencial alvo da MA.

**OBJETIVOS:**

Avaliar os efeitos cicatriciais do uso da MA em ratos tratados com 5-FU na dose de 20 mg/kg ao sétimo dia de evolução pós-operatória quanto aos parâmetros porcentagem de colágeno tipo I (maduro), viabilidade celular, densidade microvascular e formação do tecido de granulação.

**MÉTODOS:**

Utilizaram-se 32 ratos da linhagem Wistar submetidos a colotomia e colorrafia separados em quatro grupos com oito, que receberam diferentes tratamentos diariamente por via intraperitoneal até o dia do sacrifício: solução fisiológica (C), 20 mg/kg 5-FU, 20 mg/kg 5-FU e MA.

**RESULTADOS:**

O tratamento com 20 mg/kg de 5-FU, ao sétimo dia pós-operatório, induziu efeitos adversos no processo cicatricial anastomótico evidenciados pela diminuição da porcentagem de colágeno tipo I (maduro), da viabilidade celular, da densidade microvascular, da formação de crosta fibrinoleucocitária e da proliferação angiofibroblástica; o uso da MA nessas condições induziu melhora da porcentagem de colágeno tipo I (maduro).

**CONCLUSÕES:**

O tratamento com 20 mg/kg de 5-FU, ao sétimo dia pós-operatório, induziu efeitos adversos no processo cicatricial anastomótico e o uso da MA nessas condições induziu melhora da porcentagem de colágeno tipo I (maduro).

## INTRODUCTION

G astrointestinal surgeries are among the most common surgical procedures. They have transformed notably over the past two centuries, and the heroic experimental attitudes of the past have become technically refined, high-precision procedures. During this process of evolution, publications on synthesis materials have been notable, particularly those related to intestinal anastomoses^1^.

Because surgical interventions in the intestines often involve extreme and challenging conditions and are frequently the only option, many researchers have expanded knowledge on antimicrobial agents and support measures; despite wide use, they are not sufficient to entirely reduce morbidity and mortality, which continue to grow every day^21^.

One notable condition involving adverse surgical conditions is intestinal cancer, which is generally considered treatable and often curable when detected early. The primary treatment is surgery, followed by adjuvant chemotherapy with 5-fluorouracil (5-FU). Some isolated or associated causes of unsuccessful surgery have been described and include inadequate oxygen supply to the tissue involved, increased mechanical strain, nutritional deficiencies, metabolic disorders, inflammatory and infectious processes, and pharmacological agents^2,34^.

Medications that can negatively impact post-operatory evolution and the healing process include antitumoral chemotherapy agents, due to their cytostatic and antimetabolic activities. The most frequent complications in gastrointestinal surgery are peptic ulcer, intestinal ischemia, and failed anastomotic healing, which account for 3.4–12% of dehiscences^10^.

Considering that the wound healing process is dynamic and involves a variety of factors, correcting a single mechanism is unlikely to substantially improve healing of compromised wounds^30^.

Within this context, human perinatal tissue derivatives (PnD) such as the amnionic and chorionic membrane have been studied and indicated as multifaceted products offering a range of possibilities to address failures in tissue regeneration and wound closure. These products are derived from the placenta and contain numerous growth factors and cytokines that have already been proven to promote wound healing. Despite advances in understanding the role these tissues play in healing, preserving their biological activity has been a major obstacle to broader clinical use^4^.

PnD are abundant sources of human extracellular matrix proteins, growth factors, and stem cells, and have been proven useful for a wide range of therapeutic applications. They are also angiogenic, anti-inflammatory, antifibrotic, antimicrobial, and immune inducers. These tissues are often discarded as medical waste and are consequently easy and ethical to access and offer almost unlimited benefits for cost. Although some PnD such as the amniotic membrane and umbilical cord have been used in clinical practice, most continue to be significantly underutilized. Today, their most relevant application is as a source of biomaterials and cells in the nascent area of tissue engineering and regenerative medicine^22^.

There is significant scientific interest in obtaining PnD non-invasively to take advantage of their anti-inflammatory, anticancer, and antifibrotic properties. Many studies that have utilized these tissues in pre-clinical models of cutaneous wound healing have used a wide variety of animal species ranging from large animals to rodents, diabetic and non-diabetic animals, and investigated different types of lesions (full thickness wounds, burns, or skin flaps). Assessing different sources and types of PnD (placenta, umbilical cord, fetal membranes, cells, secretomes, tissue extracts), modes of administration (topical, intradermal, subcutaneous, intravenous, or intraperitoneal), and forms of application (hydrogels, synthetics or natural, biomaterials as transporters of transplanted cells, extracts, or secretomes) has been a motive for significant investments by research centers^12,17^.

Studies on the clinical applicability of the amniotic membrane (AM) have consequently been gaining importance due to its low antigenicity and activities including antimicrobial action, capacity to reduce exudate and adhesions, accelerated epithelization, reduction of local pain, and to serve as a substrate for tissue growth, justifying investment in projects that could help determine the transdisciplinary applications of this therapeutic option.

The objective of this study was to assess the healing effects when amniotic membrane was used on rats that underwent surgery to create a colonic anastomosis and also received 5-FU, specifically investigating the formation of granulation tissue, percentage of type I collagen (mature), cell viability, and microvascular density on the seventh post-operative day.

## METHODS

### Experiment design

This study was evaluated and approved by our institutional ethics board for animal research (protocol 016/16)^25^. It also followed the norms established in applicable regulation on animal experimentation (Brazilian Federal Law 11.794) and the Ethical Principles on Animal Experimentation from the Brazilian College on Animal Experimentation (COBEA-2000).

We utilized 32 Wistar rats (*Rattus norvegicus albinus, Rodentia, Mammalia*) aged 112–125 days and weighing 451.56±56.20 g, which were divided into four groups of eight animals each for the procedures (Table 1).

**Table 1:** Group organization and procedures.

Groups	n	Procedures	
		D0	D7
Control (C)	8	Treatment with saline, colostomy, and colorrhaphy	Conclusion of protocol and sample collection
5-FU	8	Treatment with 5-FU (20 mg/kg IP), colostomy, and colorrhaphy	
5FU/AM	8	Treatment with 5-FU (20 mg/kg IP), colostomy, and colorrhaphy	
AM	8	Placement of AM, colostomy, and colorrhaphy	

5FU/AM: fluorouracil+amniotic membrane; AM: amniotic membrane; 5FU: fluorouracil; IP: intraperitoneal.

The rats were screened for the presence of ectoparasites in their fur, signs of diarrhea, and cutaneous lesions. They were kept in an environment specific for laboratory animals with forced air exhaust (negative pressure), temperature controlled at 19–23°C, and light cycles automatically regulated every 12 hours. After inspection, they were separated into groups of four in polypropylene boxes, given rat feed and water *ad libitum,* and were weighed twice: once on the day the experiment began (D0) and again at the end of the protocol (D7).

### Experiment

The procedures were carried out during a single activity cycle in an environment specifically dedicated to experimental surgeries, following protocols for anesthesia in abdominal procedures.

### Preparation of the amniotic membrane

**Figure 1:**
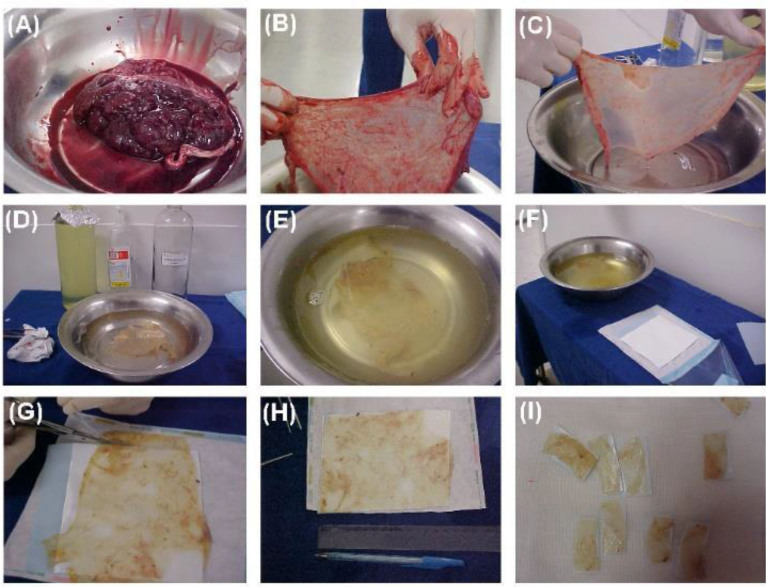
Preparation of the amniotic membrane: A) recently delivered placenta; B) manual separation of the amniotic membrane; C) disinfecting and rinsing; D, E) decellularization; F) sterilization; G, H, I) assembling the patches

The membrane was prepared as follows (Figure 1).

1. Parturient patients in the hospital’s obstetrics service who had consented to participate in the study by providing their placentas were recommended for cesarean section, having been screened for infectious diseases (HIV, hepatitis B and C, syphilis, and HTLV I and II).
2. After delivery of the placenta, it was transferred to a sterilized stainless steel container and the AM was separated manually in aseptic conditions.
3. The AM was disinfected by submersion in Dakin’s solution for 2 hours.
4. The AM was rinsed three times with rinsing solution.
5. Decellularization was performed by immersing the AM in hypotonic solution, where it was maintained for 16 hours at 2–8°C.
6. The AM was rinsed three times in rinsing solution.
7. The AM was immersed in SDS/EDTA decellularization solution for 24 hours at room temperature.
8. The AM was rinsed three times in rinsing solution.
9. The AM was immersed in DNase/RNase decellularization solution for 3 hours at 37°C.
10. The AM was rinsed one time in rinsing solution.
11. Tor sterilization, the AM was immersed in antibiotic solution for 2 hours at room temperature.
12. The AM was rinsed one time in rinsing solution.
13. AM patches were assembled.
14. In a laminar flow cabinet, on sterilized fields, the AM was fixed onto nitrocellulose paper (NitroBind 0.45 μm) which had been previously sterilized using ethylene oxide.
15. Using sterilized scissors, the fixed AM was cut into sections measuring 3.0 x 1.5 cm.
16. Four patches were placed on sterilized Petri dishes and kept at 2–8°C for no longer than 12 hours^31,33^.

### Surgical procedure

The surgery was conducted in an environment specifically dedicated to experimental procedures, and all rats were fasted for 12 hours with water *ad libitum*.

### Anesthesia

For anesthetic induction, the animals were sedated via inhalation of isoflurane in a closed circuit and weighed on an analytic scale to calculate dosage of ketamine (80 mg/kg) and xylazine (10 mg/kg), for intramuscular administration. Next, the abdomen was shaved and disinfected, and sterile fenestrated surgical drapes were placed.

### Colostomy and colorrhaphy

Using a laparotomy (median length: 3 cm), the colostomy was performed with total section of the colon at approximately 5 cm distal from the ileocecal valve, carefully preserving the colonic vessels, and end-to-end anastomosis was created in a single total plane with eight separate stitches using 6.0 nylon monofilament suture. The abdominal wall was closed with continuous 3.0 nylon suture in two planes.

In the rats in the 5-FU/AM and AM groups, a patch of AM was placed around the enteroanastomosis, and the extremities were then fixed using a 6.0 suture around the entire circumference of the anastomosis. The abdominal wall was closed with continuous 3.0 nylon suture on the musculoaponeurotic and cutaneous planes. The rats were administered dipyrone orally (20 mg/kg) along with 10 mL of saline subcutaneously in the dorsal region, and kept warm until they recovered from anesthesia. They were then returned to their labeled boxes, and for the following 24 hours were kept fasted, receiving only water supplemented with sugar (commercial) at 100 mg/mL. After this period, they were fed rat feed and water *ad libitum*.

### Treatments

The rats in the 5-FU and 5-FU/AM groups (Table 1) were administered 20 mg/kg 5-FU via intraperitonial (IP) once daily during the seven days following the surgery. The C and AM groups were administered sterile saline in an equivalent dose by weight (20 mg/kg).

### Interruption of experiment and sample collection

On the seventh day post-procedure, the experiment was interrupted and the animals were weighed and euthanized, which involved sedation by inhaling isoflurane in a closed circuit, intramuscular injection of ketamine (100 mg/kg), and cardiac puncture for exsanguination and to induce cardiac arrest. After death was confirmed, broad median laparotomy was performed and the segment of the colon with the scar was resected; these sections were placed into marked flasks for fixation in paraformaldehyde solution 4% in PBS pH 7.4 for 24 hours and then subjected to histopathological analysis.

### Assessments

The sections were analyzed to assess formation of granulation tissue, histometry and percentages of type I (mature) and type III (immature) collagen, cell viability according to proliferating cell nuclear antigen (PCNA), and microvessel density using CD34.

Weighing and histopathology of formation of granulation tissue

The rats were weighed on D0 (start of experiment) and again on D7. The histological assessments were performed by two independent pathologists on sections subjected to hematoxylin and eosin (H&E) and Harris staining to determine:

1. The intensity of the inflammatory reaction and the formation of a fibrin-leukocyte scab, from deposits of fibrin and blood cells on the surface of the wound;
2. Angiofibroblastic proliferation, by morphological assessment of neovascularization (angiogenesis); and
3. Fibroblast proliferation and reepithelization of wound borders, with special attention to the advance of the epithelium around the wound. The findings were scored 0 for lack of reaction, 1 for slight reaction, and 2 for moderate reaction. The readings were tabulated and individual values from the 2 readings (L1 and L2) for each criterion as well as the mean score for each sample were subjected to statistical assessment^27^.

### Histometry of percentage of type I and III collagen

The histological sections were stained with picrosirius red (F3BA) and magnified 400 times with polarized light. Camera images were obtained and transmitted to a colored monitor and digitized. Histometric analysis was conducted on the images using Image Pro-Plus version 4.5 software for Windows to identify collagen type based on color: red, yellow, and orange correspond to type I (mature) collagen, while green tones represent type III (immature). The findings were expressed as percent area of type I and III collagen in each field of the histological section. Results were obtained by calculating mean values from three readings of the three fields in each slide, which were then tabulated^15^.

### Cell viability as determined by proliferating cell nuclear antigen

Immunohistochemistry was used to mark proliferating cell nuclear antigen (PCNA), a marker of proliferation activity. The results were expressed as percentage of immunomarking for the PCNA antigen^9^.

### Microvessel density, as determined by CD34

The material was embedded in paraffin and 4-mm-thick sections were obtained, placed onto glass slides, and subjected to anti-CD34 antibodies.

The reactions were considered positive when a brown reaction was detected, excluding probable areas of background coloring with a nuclear reaction pattern; any brown endothelial cell or group of endothelial cells, regardless of size, which were clearly separated from other immunostained elements were considered microvessels. This analysis was performed using computed histometry. The results were expressed as tissue density of microvessels in an area of 7,578.94 mm^2^, as the mean of three readings in different microscopic fields.

### Statistical analysis

The results were expressed as mean±standard deviation. Student’s t-test with p<0.05 was used for comparisons between the groups, using GraphPad InStat software.

## RESULTS

### Weight evaluation

At the start of the experiment (D0), there were no significant differences between weights. On D7 there was a significant difference (p=0.0387) between the control group (452.6±43.5 g) and the 5-FU group (397.6±46.7 g), as well as between the 5-FU (397.6±46.7 g) and AM groups (473.9±46.9 g, p=0.0086) and between the 5-FU/AM group (395.1±58.7) and AM group (473.9±46.9 g, p=0.0149).

### Histopathological assessments of granulation tissue formation

**Figure 2:**
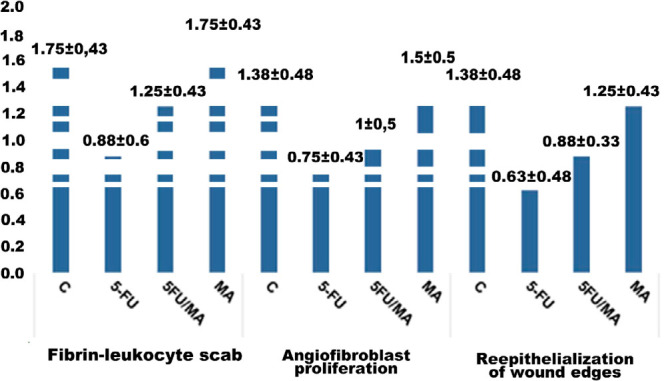
Results (mean and standard deviation) for formation of granulation tissue, fibrin-leukocyte scab, angiofibroblast proliferation, and reepithelialization of wound edges. Scoring: 0: absent; 1: slight; 2: moderate.

The samples of colon tissue were analyzed for formation of the fibrin-leukocyte scab (FLS), angiofibroblast proliferation (AFP), and reepithelialization of wound edges (RE). As shown in Figure 2, the mean score for formation of the fibrin-leukocyte scab (FLS) in the control group was significantly higher than in the 5-FU (0.0073) and 5-FU/AM groups (0.04858). For angiofibrin proliferation (AFP), the mean score for the control group was significantly higher than the 5-FU group (0.0233); the average score for the AM group was significantly higher (0.0095) than the 5-FU group. As for reepithelialization of wound edges (RE), the mean score for the control group was significantly higher than for the 5-FU group (0.01168) and 5-FU/AM group (0.0405). The average score for the AM group was significantly higher than for the 5-FU group (p=0.0233).

### Histometric assessment of percentage of type I (mature) and (immature) collagen

Collagen formation differed significantly between the control group and 5-FU group (p=0.0055, p<0.05). The mean score for type I collagen in the group treated with 5-FU was significantly lower than for the 5-FU/AM group (p=0.0154, p>0.05) and AM group (p=0.0368, p>0.05).

### Assessment of cell viability via proliferating cell nuclear antigen

Significant differences were found between the control group and the 5-FU group (p=0.0012, p<0.05) and the 5-FU/AM group (p=0.0028, p<0.05). The mean for positive PCNA in the group that received 5-FU was significantly lower than in the AM group (p=0.0008), and this value for the 5-FU/AM group was also significantly lower than for the AM group (0.0019, p<0.05).

### Assessment of microvessel density, as determined by CD34

Significant differences were found between the control group and the 5-FU group (p=0.0001, p<0.05) and the 5-FU/AM group (p=0.0001, p<0.05). The mean for positive CD34 in the group that received 5-FU was significantly lower than in the AM group (p=0.0006, p<0.05) and this value for the 5 FU/AM group was also significantly lower than for the AM group (0.0005, p<0.05).

## DISCUSSION

The experimental model we used was proposed by Uludag et al. in 2009^32^ to investigate whether covering colonic anastomoses with AM improved healing of the anastomoses in spite of the toxic effects of immediate administration of 5-FU. These scientists used 120 Wistar rats. Our study differed in the number of rats used (n=32), the day that the experiment was interrupted (D7), the final weighing of the animals, euthanasia and collection of the colonic anastomoses, and histopathological studies of granulation tissue formation via H&E staining, histometry of the percentage of type I and III collagen using picrosirius red, immunohistochemical assessment of cell viability by qualifying the percentage of PCNA, and immunohistochemical assessment of microvessel density using the CD34 angiogenesis marker.

We selected 5-FU for this study as a factor with potential negative impacts on the healing process in the colon because this drug was initially described as non-selective for tumor cells, exercising its antimetabolic effects on various tissues and cells involved in the process of anastomotic healing in young and adult animals^8^. The dose of 5-FU we utilized (20 mg/kg) has already been confirmed as causing reduced capacity for collagen synthesis in anastomoses, possibly by reducing the number of fibroblasts and consequent direct inhibition of collagen synthesis. This treatment has been determined to significantly compromise healing of the colonic operative wound, demonstrated in a significant reduction in anastomosis bursting pressure and breaking strength^33^.

Another experimental rat study assessing the effects of granulocyte-macrophage colony-stimulating factor (GM-CSF) on colonic anastomoses after post-operative intraperitoneal chemotherapy using 5-FU (20 mg/kg) proved that macrophage activity decreased and wound healing was compromised, and that healing improved when GM-CSF was used^14^.

The toxic effects of 5-FU on the rats in this study were initially seen in weight loss in the 5-FU groups (397.6±46.7 g) at D7 compared to the control group (452.6±43.5 g, p=0.0387), which is in line with findings from similar studies^19,20,23,24^.

### Assessment of the healing process

The stages of healing for intestinal anastomoses are similar to those in other tissues, in which the initial inflammatory response occurs over 1–3 days, followed by the proliferative phase at 3–14 days, when granulation tissue is formed, along with greater angiogenic stimulation and collagen synthesis (day 7), followed by the remodeling phase, with deposit and maturation of collagen followed by scar maturation^1,13^. The final part of the proliferative phase is the formation of granulation tissue. Fibroblasts and endothelial cells are the main cells involved in this phase: fibroblasts from neighboring tissue migrate to the wound, but must be activated to awaken from their quiescent state^18^.

In this study, comparison of the formation of granulation tissue between the control group and the 5-FU group found significantly less formation of the fibrin-leukocyte scab (p=0.00737, p<0.05) and angiofibroblast proliferation (p=0.02330, p>0.05) in the treated rats, but no significant difference was seen in reepithelialization of wound edges (p=0.2782, p>0.05), confirming the deleterious effects of 5-FU in the healing process.

These results are corroborated by findings from other authors who also verified poorer healing in the colon and other tissues. In rats treated with 5-FU, major loss of strength in the anastomoses was observed, measured by breaking strength in the rats that underwent resection of the ileum and colon, with concomitant reduction of collagen in the wound^25^. The authors concluded that anastomosis repair in rats proceeds normally under ideal conditions, but is altered in the presence of 5-FU^17^. In another experimental study of rats that also underwent resection of the colon and colorrhaphy, the rate of anastomosis dehiscence and formation of adhesions increased, bursting pressure decreased, along with infiltration rates of inflammatory cells, neoangiogenesis, fibroblast activity, collagen deposit, and hydroxyproline levels, leading to increased levels of malondialdehyde^33^.

5-FU has already been used to induce mucositis in rats. Poorer repair of the oral mucosae has been described in rats treated with 5-FU compared to an untreated control group, also demonstrating the action of 5-FU on oral mucosa tissues via the histopathological indicators CD4, CD8, PCNA, and cell proliferation indicator VEGF^20^.

Intraperitoneal application of 5-FU at 20 mg/kg were also found to produce worst outcomes in assessments of anastomosis bursting pressure, levels of hydroxyproline in the tissue, edema formation in the tissue, necrosis, and collagen formation^32^.

In our assessment of percentages of type I and III collagen, poorer outcomes were seen in the 5-FU group (73.85±6.1) compared to the control group (83.4±4.73, p=0.0055, p<0.05), the 5-FU/AM group (82.07±4.99, p=0.0154, p>0.05), and the AM group (80.47±4.51, p=0.0368, p>0.05). To assess collagen levels in the experiment, spectrophotometric methods were used to measure the levels of hydroxyproline, and histochemical assessment using picrosirius red staining^29^.

The collagen molecule is composed of three polypeptide chains arranged in a triple helix containing proline, hydroxylysine, and hydroxyproline. Proline and hydroxyproline stabilize these chains, with hydroxyproline accounting for 14% of collagen^6^. As of this writing, 28 different types of collagen have been identified in mammals; the four most common are numbered I to IV. They differ in terms of chemical composition, mode of association between the molecules, functions, and diseases that their malformation or excessive or insufficient production may cause. In intact tissue, type I collagen predominates, accounting for 80–90%, and the remaining 10–20% are type III. In the gastrointestinal tract, the submucosal layer contains the most collagen compared to the other layers^1^. The behavior and dynamics of collagen can be demonstrated practically, as in the case of anastomosis healing; recent scars have more type III collagen than type I, since this latter type predominates in the later phases of healing^26^.

In 1978, Junqueira et al. confirmed that picrosirius red staining specifically shows collagen in different tissues. Under polarized light, the thicker and more interconnected fibers appear as reddish-orange and are designated type I (mature). The fibers that are more tapered and less interconnected are greenish, and are considered type III collagen (immature)^15^.

In other experimental studies like ours involving rats subjected to colostomy and colorrhaphy, improved levels of collagen deposits were seen in colon anastomoses overlaid with AM, as determined by measuring the levels of hydroxyproline in the tissue^11^.

As for quantification of viable cells by PCNA, the 5-FU group showed a significant decrease (p=0.0012, p<0.05) compared to the control group, which is in line with the results for formation of the fibrin-leukocyte scab and reepithelialization of wound edges in assessing the formation of granulation tissue. This group also presented significantly lower results (p=0.0001, p<0.05) than the control group for quantification of microvessel density in the scar, quantified by immunohistochemical testing for the CD34 receptor, which is in line with the results for angiofibroblast proliferation in the formation of granulation tissue.

In the groups that received AM patches, cell viability was not notably improved compared to the 5-FU (60.63±5.59) and 5-FU/AM (63±16.23, p=0.7862, p>0.05) groups. Similar findings were seen in the assessments of microvessel density, when compared to the 5-FU (2.63±1.87) and 5-FU/AM (2.5±1.94, p=0.9038, p>0.05) groups.

Use of decellularized AM, with minimal negative effects on the components of the extracellular matrix, is very important to avoid graft rejection and presents better performance^5^. As of this writing, various techniques for decellularizing the AM have been proposed, and are mainly based on enzymatic procedures associated with the action of detergents, with distinct results according to the technique used. Here we used a combination of the detergent effects of sodium dodecyl sulfate and enzymatic action of RNAse and DNAase, since the human origin of the AM would certainly induce an active immune response in rats due to the phylogenetic distance between the two species. The decellularization protocol demonstrated reliable histological evidence in removing cells or fragments, minimizing antigenicity with minimal damage to the components of the extracellular matrix. Other benefits of this current decellularization protocol based on detergent and enzymatic action include affordability, simplicity, speed, and safety^16^. The AM has been explored as a very promising biologically based product in humans, particularly for wound healing applications on the skin and cornea; its properties offer potential for treating various cutaneous wounds such as bedsores, burns, diabetic ulcers, osseous lesions, pelvic floor disorders, and venous ulcers^7,16^. Its most important property is its cytobiocompatibility, since its structure is very similar to normal skin, composed of proteins in the extracellular matrix^3,5,7,28,35,36^.

The detergent-based process of decellularizing the AM we utilized in this study is considered a reliable protocol for this purpose, since it removes cells and fragments, and there is no need to histologically prove decellularization took place by this method.

## CONCLUSIONS

Using the AM in the process of anastomosis healing in rats treated with 20 mg/kg of 5-FU led to improved percentage of type I (mature) collagen on day seven after surgery.
